# Overexpression of MUC1 Induces Non-Canonical TGF-β Signaling in Pancreatic Ductal Adenocarcinoma

**DOI:** 10.3389/fcell.2022.821875

**Published:** 2022-02-14

**Authors:** Mukulika Bose, Priyanka Grover, Alexa J. Sanders, Ru Zhou, Mohammad Ahmad, Sophia Shwartz, Priyanka Lala, Sritama Nath, Mahboubeh Yazdanifar, Cory Brouwer, Pinku Mukherjee

**Affiliations:** ^1^ Department of Biological Sciences, UNC Charlotte, Charlotte, NC, United States; ^2^ Department of Bioinformatics, UNC Charlotte, Charlotte, NC, United States

**Keywords:** MUC1—mucin 1, pancreatic ductal adenocarcinoma, non-canonical pathways, JNK (c-Jun N-terminal kinase), TGF-beta

## Abstract

Pancreatic ductal adenocarcinoma (PDA) is one of the most lethal human cancers. Transforming Growth Factor Beta (TGF-β) is a cytokine that switches from a tumor-suppressor at early stages to a tumor promoter in the late stages of tumor development, by yet unknown mechanisms. Tumor associated MUC1 is aberrantly glycosylated and overexpressed in >80% of PDAs and is associated with poor prognosis. MUC1 expression is found in the early stages of PDA development with subsequent increase in later stages. Analysis of human PDA samples from TCGA database showed significant differences in gene expression and survival profiles between low and high MUC1 samples. Further, high MUC1 expression was found to positively correlate to TGF-βRII expression and negatively correlate to TGF-βRI expression in PDA cell lines. We hypothesized that MUC1 overexpression induces TGF-β mediated non-canonical signaling pathways which is known to be associated with poor prognosis. In this study, we report that MUC1 overexpression in PDA cells directly activates the JNK pathway in response to TGF-β, and leads to increased cell viability via up-regulation and stabilization of c-Myc. Conversely, in low MUC1 expressing PDA cells, TGF-β preserves its tumor-suppressive function and inhibits phosphorylation of JNK and stabilization of c-Myc. Knockdown of MUC1 in PDA cells also results in decreased phosphorylation of JNK and c-Myc in response to TGF-β treatment. Taken together, the results indicate that overexpression of MUC1 plays a significant role in switching the TGF-β function from a tumor-suppressor to a tumor promoter by directly activating JNK. Lastly, we report that high-MUC1 PDA tumors respond to TGF-β neutralizing antibody *in vivo* showing significantly reduced tumor growth while low-MUC1 tumors do not respond to TGF-β neutralizing antibody further confirming our hypothesis.

## Introduction

Pancreatic Cancer is currently the third leading cause of cancer-related deaths in the United States (http://pancreatic.org/). It has been projected to become the second leading cause of cancer-related deaths in the US, surpassing colorectal cancer by the year 2030 (http://pancreatic.org/). About 95% of pancreatic cancers are pancreatic ductal adenocarcinomas (PDA) with patients demonstrating a median survival rate of less than 6 months and a 5-year survival rate of 9% in the US (McGuigan et al., 2018). In the US, the rate of new pancreatic cancer cases is 13.1 per 100,000 people per year and the mortality rate is 11.0 per 100,000 people per year (Howlader et al., 2017). Therefore, it has a mortality rate that nearly matches its incidence rate.

The transforming growth factor beta (TGF-β) signaling pathway belongs to a large superfamily that primarily consists of TGF-β (including isoforms of TGF-β1, 2, and 3), bone morphogenetic proteins, activins, and inhibins (Namwanje and Brown, 2016). This family of growth factors activates many biological signals, such as cell growth, apoptosis, differentiation, immune response, angiogenesis, and inflammation (Cárcamo et al., 1994; Shi, 2001; Isabel et al., 2014). Deregulation of the TGF-β pathway can lead to cancer, among other ailments (Colak and ten Dijke, 2017). In normal environments and early cancers, TGF-β regulates epithelial cells as a tumor suppressor by controlling cell cycle and inducing apoptosis. However, in certain cases, once the cancer is established, a switch occurs and TGF-β becomes a tumor promoter. TGF-β induces invasion and migration and eventually leads to epithelial-to-mesenchymal transition (EMT) (Massagué, 2008). This process helps facilitate the migration and invasion of cancer cells to distant locations leading to metastasis, the major cause of cancer-related deaths (Mittal, 2018).

The canonical TGF-β signaling is initiated by the binding of a TGF-β cytokine to a pair of specific transmembrane receptors, TGF-βRI and TGF-βRII (Massagué and Chen, 2000). This activates the cytoplasmic serine/threonine kinase domains of the TGF-β receptors (Wrana et al., 1994), which leads to further activation downstream. In normal environments, TGF-β binds to its specific receptors TGF-βRII and TGF-βRI, in sequence. This leads to the phosphorylation of SMAD2/3 via the cytoplasmic Serine/Threonine kinase domain of TGF-βRI (Tsukazaki et al., 1998). SMAD2 has been identified as a tumor suppressor and mediator of the antiproliferative TGF-β and activin responses (Eppert et al., 1996). SMAD2/3 trilocalizes with SMAD4 (Attisano and Wrana, 2002). This leads the heterotrimer complex to the nucleus to induce transcriptional changes that influence cell regulation (Attisano and Wrana, 2002) (Massagué and Wotton, 2000). However, frequent alterations and changes in the TGF-β pathway occur in cancer, especially in PDA. Dysregulated TGF-β signaling activates ERK1/2 and JNK (Zhang, 2009) leading to an increase in aggressive cancer characteristics, such as growth, invasion, migration, and metastasis (Lee et al., 2007).

Mucin-1 (MUC1) is a Type I transmembrane glycoprotein that influences tumor progression and metastasis in PDA (Nath and Mukherjee, 2014). Tumor-associated MUC1 is overexpressed and aberrantly glycosylated in more than 80% of PDA cases (Tinder et al., 2008; Kufe, 2009; Roy et al., 2011; Nath and Mukherjee, 2014; Zhou et al., 2016). In normal environments, MUC1 is expressed on the apical surface of ductal cells to provide a protective barrier (Kato et al., 2017). However, upon tumorigenesis MUC1 expression is no longer restricted to the apical surface. At this point, MUC1 glycosylation decreases and the protein becomes overexpressed across the cell surface, placing it into the close vicinity of many growth factor receptors (Kufe, 2009). MUC1 oncogenic signaling, which plays an important role in increased metastasis and invasion, is promoted through the cytoplasmic tail (MUC1-CT). The MUC1-CT is a highly conserved 72-amino acid long domain containing seven tyrosine residues that are phosphorylated by non-receptor tyrosine kinases, such as c-SRC (Thompson et al., 2005; Singh and Hollingsworth, 2006). Importantly, MUC1 modulates TGF-β signaling in PDA cell lines that were engineered to overexpress MUC1. We established that TGF-β signaling required tyrosine phosphorylation of the MUC1-CT via tyrosine kinase c-SRC (Grover et al., 2018). Here we deepen our understanding of MUC1 regulation of TGF-β signaling in PDA cells that are genetically varied and that express varying levels of endogenous MUC1. We establish that the level of MUC1 expression plays a definitive role in inducing the TGF-β -induced non-canonical pathway. In the presence of high levels of MUC1, TGF-β activates the JNK pathway, and enhances cell viability by activating and stabilizing c-Myc. In PDA cells with low levels of MUC1, TGF-β induces growth inhibition. Taken together, our study suggests a novel role of MUC1 in TGF-β signaling in PDA. The *in vivo* data demonstrates that high-MUC1 PDA responds well to the TGF-β neutralizing antibody while low MUC1 PDA does not.

## Results

### Differential Gene Expression Profiles in TGF-β, MAPK and BMP Pathways in High Versus Low MUC1 PDA Samples

**FIGURE 1:**
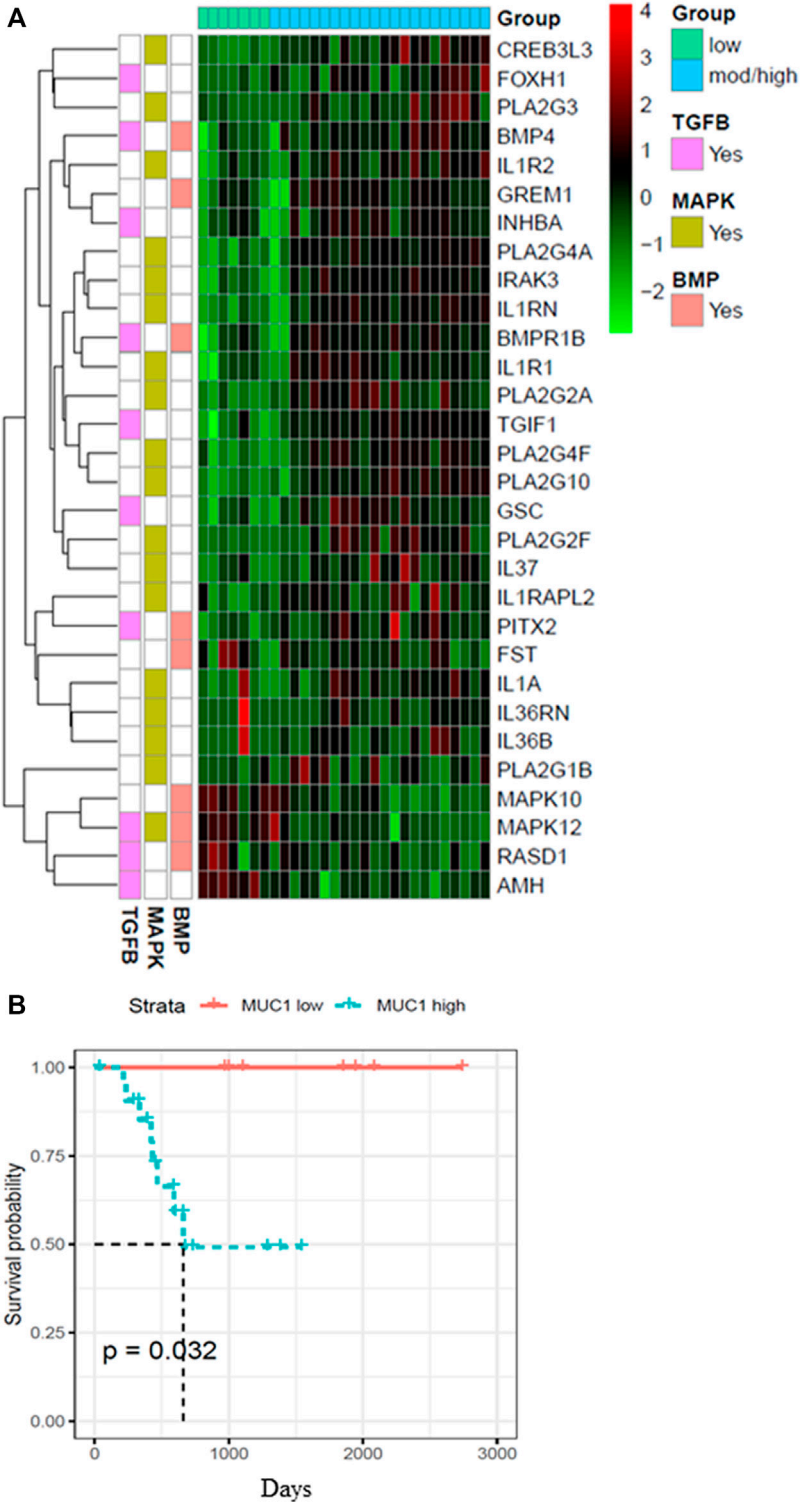
Heatmap showing top 30 differentially expressed genes in high/moderate vs low MUC1 PDA samples from TCGA. **(A)** Top panel shows the color key for MUC1 expression in the 29 PDA samples. Right hand side shows the color key histogram for expression levels of each gene named on the right. Left hand side color key shows the genes associated with each of the three pathways in pink (TGF-β), green (MAPK) and peach (BMP). Genes with a false discovery rate adjusted *p* < 0.05 are shown. **(B)** Kaplan-Meier curve for overall survival (OS) in the 29 PDA patients from TCGA in low (blue) vs high/moderate (red) groups are shown.

Since the role of MUC1 in oncogenesis is well known, we utilized the TCGA database to look for differences in the gene expression profiles between samples with low MUC1 and moderate/high-MUC1 expression (Figure 1A). Out of >4,000 genes that were differentially expressed (data not shown), the top 30 genes that are involved in the TGF-β, MAPK and BMP pathways were selected to create the heatmap since these pathways are known to be regulated by TGF-β. Several known transcription factors like CREB3L3, FOXH1, PLA2G3, BMP4 as well as immune related genes such as the IL1R1 and IL1R2 were upregulated in high MUC1 samples which are all associated with increased epithelial to mesenchymal transition (EMT) and poor survival (Hamada et al., 2007; Zhang J et al., 2021; Zhang M et al., 2021). It is highly interesting to note that GREM 1, a key pro-fibrogenic factor in PDA (Davis et al., 2022) is upregulated in MUC1-high PDA and downregulated in MUC1-low PDA. Furthermore, INHBA, a ligand for TGF-β and associated with tumorigenesis (Cao et al., 2004) is upregulated in high-MUC1 and downregulated in low-MUC1 PDA. In contrast, we found downregulation of MAPK10, MAPK12, RASD1 and AMH in MUC1-high and upregulation of the same genes in MUC1-low PDA. Downregulation of these genes correlate with poor survival (human protein atlas). These data indicate the differential TGF-β signaling in high versus low MUC1 PDAs where TGF-β predominantly promotes oncogenic signaling in high-MUC1 PDA. The protein-protein interaction networks of these 30 genes in low vs high MUC1 samples are shown in Supplementary Figure S1, further confirming the functional role of MUC1 in TGF-β associated oncogenic signaling. Thus, it was not surprising that MUC1 expression had a significant correlation with poor overall survival (OS) in PDA patients (Figure 1B).

### High -MUC1 Expression in PDA Cells Positively Correlates to TGF-βRII and Negatively Correlates to TGF-βRI Levels

**FIGURE 2:**
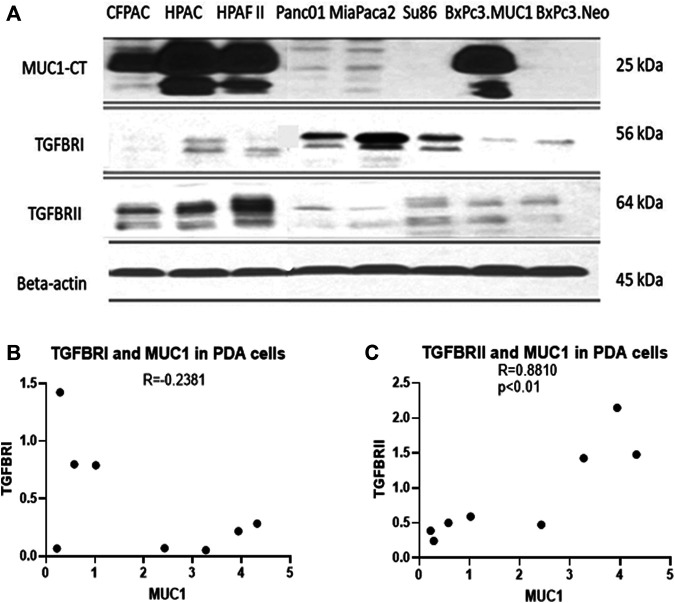
High MUC1 expression in PDA cells positively correlates to TGF-βRII and negatively correlates to TGF-βRI levels. **(A)** The expression of MUC1-CT, TGF-βRI, TGF-βRII, and endogenous loading control β-actin in a panel of PDA cell lines, determined by Western blot. **(B)** Densitometric analysis of MUC1 expression versus TGF-βRI expression shows a negative correlation (Spearman’s correlation coefficient r = -0.2381, NS). **(C)** Densitometric analysis of MUC1 expression versus TGF-βRII expression shows a significantly positive correlation (Spearman’s correlation coefficient r = 0.8810, *p* = 0.0072).

Several studies have shown that MUC1 overexpression in PDA is linked to enhanced growth and metastasis (Besmer et al., 2011; Roy et al., 2011; Sahraei et al., 2012). Since TGF-β signaling starts with binding of TGF-β to its receptors followed by activation of the same, we investigated the correlation between MUC1 and TGF-β receptor expression levels in select PDA cell lines. We selected a panel of human PDA cell lines with varying levels of MUC1 expression (Figure 2A), and assessed the expression of MUC1, TGF-βRI and TGF-βRII by Western Blotting (Figure 2B). Results were profound. All high-MUC1 PDA cells (CFPAC, HPAC, HPAFII, and BxPC3. MUC1) expressed lower levels of TGF-βRI and significantly higher levels of TGF-βRII as compared to the low-MUC1 PDA cells (Panc01, MiaPaca2, Su86.86, and BxPC3. Neo). By statistical analysis, these results show a negative correlation (-0.2381) between MUC1 and TGF-β RI expression (Figure 2B) and a significantly high overall positive correlation (0.8810 with a *p* value of <0.01) between MUC1 and TGF-β RII expression (Figure 2C).

### TGF-β Induces Activation of the Non-Canonical Signaling in High MUC1 PDA Cells

**FIGURE 3:**
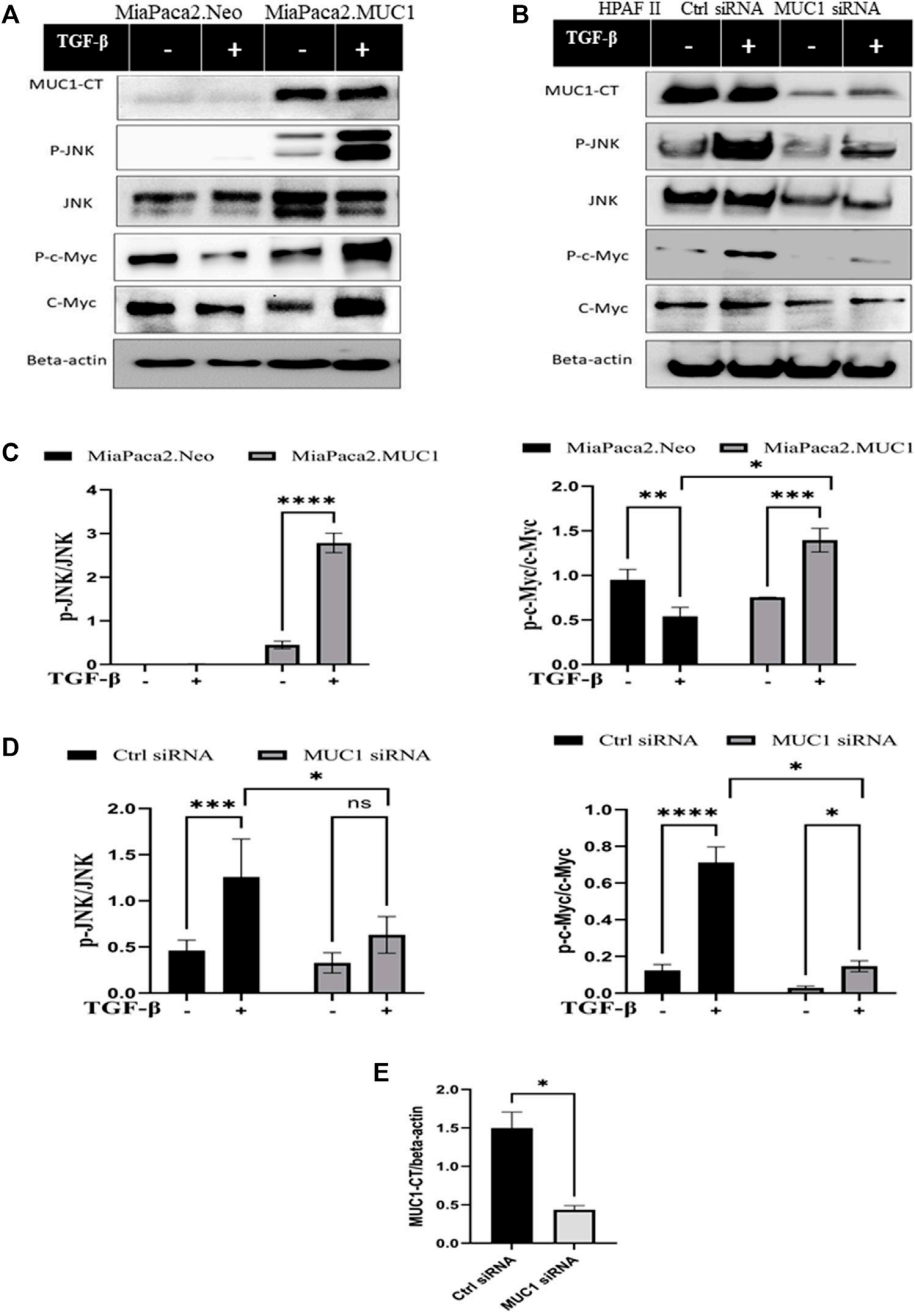
Overexpression of MUC1 leads to increased phosphorylation of JNK and c-Myc and knockdown of MUC1 reduces phosphorylation of JNK and c-Myc. **(A)** Western blot expression of phosphorylation of JNK and c-Myc compared to total JNK and total c-Myc in MiaPaca2 vs MiaPaca2. MUC1 cells in response to 10 ng/ml of TGF-β at 10 min. **(B)** Western blot expression of phosphorylation of JNK and c-Myc compared to total JNK and total c-Myc in HPAFII cells treated with control siRNA vs MUC1 siRNA in response to 10 ng/ml of TGF-β at 10 min. **(C)** Densitometric analysis of fold change of expressions of pJNK/Total JNK and p-c-Myc/Total c-Myc normalized to endogenous β-actin is presented in MiaPaca2 cells. **(D)** Densitometric analysis of fold change of expressions of pJNK/Total JNK and p-c-Myc/Total c-Myc normalized to endogenous β-actin is presented in HPAFII cells. **(E)** Knockdown efficiency of MUC1 in HPAFII after 72 h of siRNA treatment. Data are presented as means ± SEM of n = 3; Unpaired Student’s t-test and one-way ANOVA were used to analyze the differences between treatment groups. *p < 0.05, **p < 0.01, ***p < 0.001, ****p < 0.0001.

Since the receptor levels are associated with the canonical and non-canonical TGF-β signaling pathways, we examined changes in phosphorylation of JNK and c-Myc in response to TGF-β in high versus low-MUC1 PDA cell lines (HPAFII and MiaPaca2 respectively). We overexpressed MUC1 in MiaPaca2 cells and downregulated MUC1 in HPAFII cells. We observed profound changes in JNK and c-Myc activation. MiaPaca2. MUC1 cells (MiaPaca2 transfected with full-length MUC1) showed significant increase in pJNK and p-c-Myc in response to TGF-β. Interestingly, there was no activation of pJNK and reduced activation of p-c-Myc in the MiaPaca2. Neo cells (MiaPaca2 transfected with empty vector) in response to TGF-β as compared to MiaPaca2. MUC1 cells (Figures 3A,C). In MiaPaca2. Neo, there was no phosphorylation of JNK even at 20 min (Supplementary Figure S2) post TGF-β treatment, however, in MiaPaca2. MUC1, there was phosphorylation of JNK starting at 10 min post TGF-β treatment (Figures 3A,C). Phosphorylation of c-Myc at Ser62 is a marker of stability of c-Myc (Wang et al., 2011). In response to TGF-β, there was a decrease in both phosphorylated Ser62 and total c-Myc in MiaPaca2. Neo cells but increased p-c-Myc and c-Myc in MiaPaca2. MUC1 cells. These results corroborate the hypothesis that TGF-β slows proliferation in low MUC1 PDA cells but promotes the same in MUC1 high cells (Figures 3A,C). In contrast, HPAFII showed high levels of pJNK and JNK as well as p-c-Myc in response to TGF-β, both of which were significantly reduced when MUC1 was knocked down using specific siRNA (Figures 3B,D). There is some background phosphorylation of c-Myc in HPAFII. MUC1 siRNA because the MUC1 KO is not 100%, however, it is clear that c-Myc phosphorylation has reduced significantly even at ∼70% KD (Figures 3D,E). Taken together, this confirms that MUC1 is associated with activation of the JNK pathway, as found in previous studies (Li et al., 2015). In this study we correlate this activation with response to TGF-β.

### Differential Viability of High and Low MUC1 PDA Cells in Response to TGF-β

**FIGURE 4:**
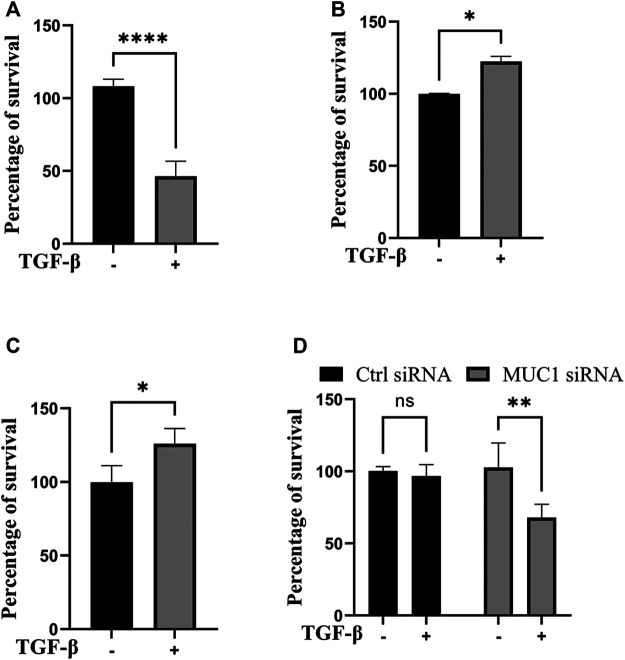
TGF-β exposure increases viability in cells with high MUC1 and reduces viability in low MUC1 PDA cells. MTT cell viability assay on **(A)** MiaPaca2.Neo cells with 10 ng/ml of TGF-β for 48 h. **(B)** HPAFII and **(C)** MiaPaca2. MUC1 cells with 10 ng/ml of TGF-β for 72 h. **(D)** HPAFII treated with control or MUC1 siRNA for 72 h followed by treatment with 10ng/ml of TGF-β for 24 h. All data are shown as means ± SEM of n = 3. Unpaired t-test was performed to compare between treated and untreated cells for each one of experiments A-C and two-way ANOVA was used to compare between untreated and treated in HPAFII.controlsiRNA and HPAFII.MUC1siRNA. *p < 0.05, **p < 0.01, ***p < 0.001, ****p < 0.0001.

Since JNK signaling promotes cell growth (Tam and Law, 2021), we next assessed cell viability *in vitro* in high-MUC1(HPAFII) and low-MUC1 (MiaPaCa2) cells in response to TGF-β. TGF-β treatment significantly reduced the viability of MiaPaca2 in 48 h and HPAFII. MUC1siRNA in 24 h, and increased the viability of HPAFII and MiaPaca2. MUC1 cells after 72 h (Figures 4A–D). Furthermore, this effect was enhanced with 96 h of incubation (Supplementary Figure S3A, B). This is also in line with our previously published work where we showed that treatment with TGF-β led to increased apoptosis in MUC1-low PDA cells (Grover et al., 2018).

### TGF-β Neutralizing Antibody Treatment Significantly Dampens High-MUC1 Tumor Growth but has No Significant Effect on Low MUC1 Tumors *in vivo*

**FIGURE 5:**
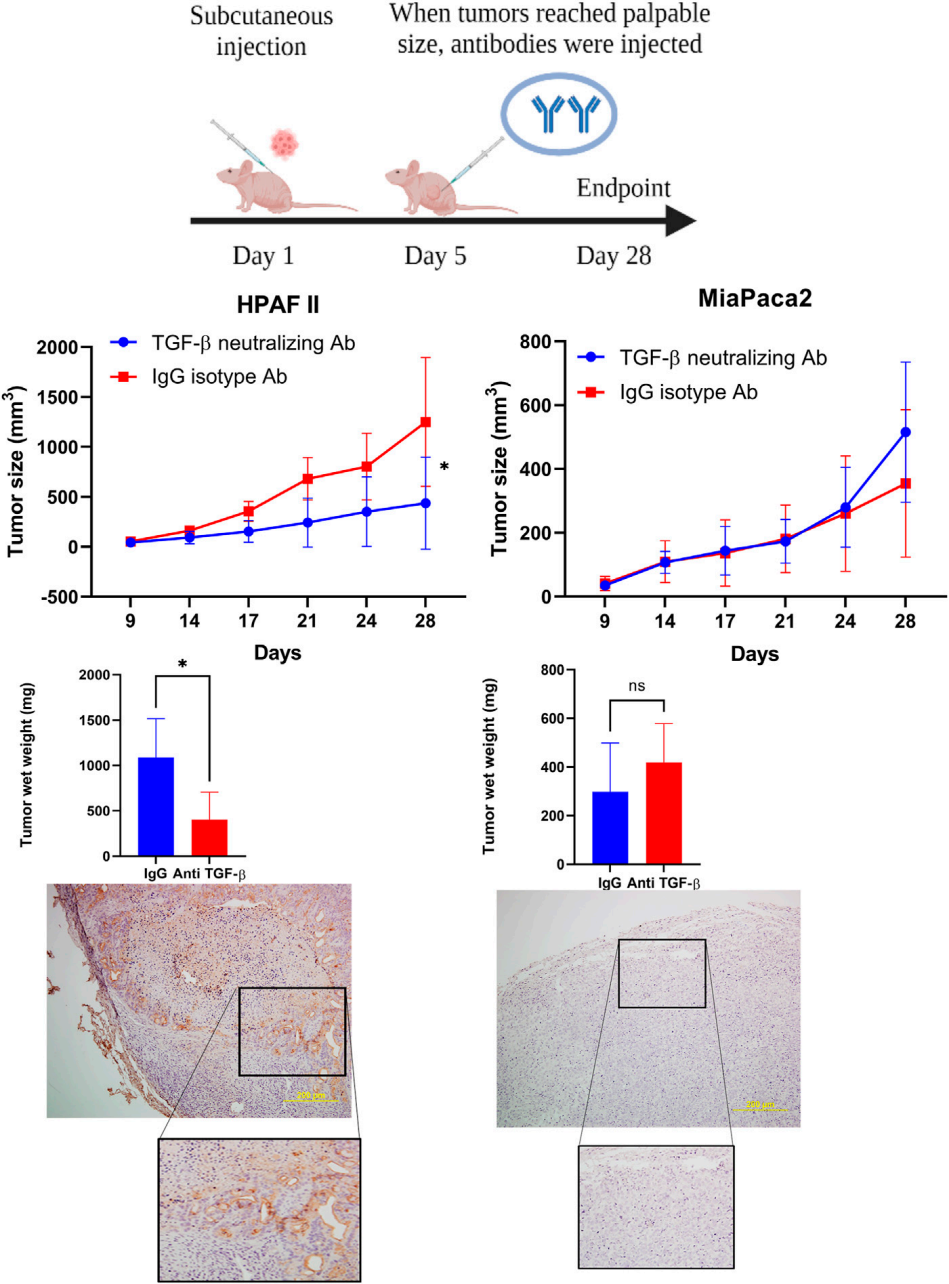
TGF-β neutralizing antibody treatment significantly reduced high-MUC1 (HPAFII) but not low MUC1 (MiaPaca2) tumor growth *in vivo*. **(A)** A schematic of the xenograft study showing the treatment with control IgG and anti-TGF-β antibody (20 ug/100 ul per mouse). **(B)** On the left: Tumor growth of HPAFII (n = 5 for TGF-β neutralizing Ab and n = 4 for IgG isotype) is shown. On the right: Tumor growth of MiaPaca2 (n = 6 for both groups) is shown. Tumor growth was determined biweekly by caliper measurements and tumor size in mm^3^ is plotted. **(C)** Wet weight of HPAFII tumors (left) and MiaPaca2 tumors (right) respectively are shown. Two-way ANOVA was used to compare between the different treatment groups. *p <0.05, NS: non-significant. **(D)** Immunohistochemistry showing expression of MUC1 in MiaPaca2 (left) and HPAFII (right) tumors.

Given that our data showed that high level of MUC1 promotes non-canonical signaling pathway in response to TGF-β, we hypothesized that treatment with anti-TGF-β neutralizing antibody would hamper growth of high-MUC1 but not of low MUC1 tumors *in vivo*. Athymic Nude-Foxn1nu mice were inoculated with HPAFII or MiaPaCa2 cells subcutaneously. Once tumors were established, mice were injected intra-tumorally with either control IgG or TGF-β neutralizing antibody three times a week for 2 weeks (Figure 5A). We observed significant reduction in tumor growth (Figure 5B) and tumor wet weight (Figure 5C) when HPAFII tumor bearing mice were treated with TGF-β antibody as compared to those in the control IgG group. In contrast, MiaPaCa2 tumors did not respond to TGF-β neutralizing antibody treatment (Figure 5B). Even though it’s not statistically significant, there was a trend of increased tumor burden in TGF-β antibody treated MiaPaca2 tumors than the IgG treated group (Figure 5C). Since TGF-β acts as a tumor promoter in high-MUC1 PDA cell lines and as a tumor suppressor in low-MUC1 PDA cell lines, it makes sense that neutralizing TGF-β in high-MUC1 cells (HPAFII), reduced tumor growth as the tumor promoting effect of TGF-β was inhibited by the antibody. On the other hand, in low-MUC1 cells (MiaPaca2), TGF-β serves as a tumor suppressor and therefore when the tumor suppressing effect of TGF-β was neutralized, tumor growth was increased, albeit not significantly. The MUC1 expression in MiaPaca2 and HPAFII tumors are shown in Figure 5D The TGF-β expression levels in MiaPaca2 and HPAFII tumors at endpoint are shown in Supplementary Figure S4A. The treatment did not have any adverse effect on the body weight of the mice (Supplementary Figure S4B).

## Discussion

MUC1 is a very interesting molecule. In normal cells, it provides protection against infection and inflammation, however, in cancer cells, MUC1 is aberrantly glycosylated and overexpressed and increases inflammation and aids oncogenesis (Bose and Mukherjee, 2020; Chen et al., 2021; Sahraei et al., 2021). In 2009, the National Cancer Institute had ranked MUC1 as the second most targetable antigen out of 75 to develop cancer vaccines (Cheever et al., 2009). MUC1 is overexpressed in more than 80% of PDA cases (Nath and Mukherjee, 2014) and TGF-β signaling plays an important oncogenic role in majority of cancers especially in PDA (Massagué, 2008). The data presented here demonstrates that MUC1 regulates TGF-β signaling and function in PDA cells. In our previous study, we reported that overexpression of MUC1 in BxPC3 cells (BxPC3. MUC1) enhanced the induction of epithelial to mesenchymal transition, and invasive potential in response to TGF-β while resisted TGF-β induced apoptosis by downregulating levels of cleaved caspases. We also showed that mutating the seven tyrosines in MUC1-CT to phenylalanine reverses the TGF-β induced invasiveness (Grover et al., 2018).

To further assess the clinical significance of MUC1 and TGF-β signaling crosstalk, we first analyzed the gene expression profiles in high and low MUC1 PDA patient samples registered in the TCGA dataset. We analyzed 29 RNA-seq samples which were from all stages, reducing the stage bias in the analysis (Supplementary Table S1). We found >4,000 genes differentially expressed (data not shown), however, we selected to further study the genes that were a part of the MAPK/JNK, BMP and TGF-β pathways, because these pathways are known to be highly regulated by TGF-β. The top 30 genes that were found to be differentially expressed in low vs high MUC1 tumors have significant roles in inflammation, cancer progression and OS (Figures 1A,B). Most of the genes upregulated in high/moderate MUC1 samples (Figure 1A) are known to be involved in increased proliferation and induction of epithelial to mesenchymal transition (EMT) or worse OS in the human pancreatic cancer, for example, CREB3L3 (CAMP Responsive Element Binding Protein three Like 3) (https://www.genecards.org/cgi-bin/carddisp.pl?gene=CREB3L3), FOXH1(Forkhead box protein H1) (Zhang J et al., 2021), BMP4 (Bone morphogenetic protein 4) (Gordon et al., 2009), IL1R2, receptor to IL-1, a cytokine known to be secreted by pancreatic cancer cells (Arlt et al., 2002; Matsuo et al., 2004; Rückert et al., 2010), GREM1 (Gremlin 1), a key pro-fibrogenic factor known to increase pancreatic inflammation and progression (Davis et al., 2022), INHBA (Inhibin βA), a ligand of the TGF-β superfamily known to be overexpressed in pancreatic cancer (Cao et al., 2004) (Mouti and Pauklin, 2021), BMPR1B, the bone morphogenetic protein (BMP) receptor family of transmembrane serine/threonine kinases (https://www.cancer-genetics.org/BMPR1B.html), TGIF1 (TGF-B Induced Factor Homeobox 1) (Razzaque and Atfi, 2020), GSC (Goosecoid Homeobox) (Kang et al., 2014) (Xue et al., 2014) and PITX2 (paired-like homeodomain transcription factor 2, also known as pituitary homeobox 2), (Xu et al., 2015) (Aubele et al., 2017; Jiang et al., 2019).

Genes that were upregulated in low-MUC1 PDA samples (MAPK12, RASD1, and AMH) were found to be favorable for OS in pancreatic cancer (Human protein atlas) (Figures 1A,B). Specifically, RASD1 (Ras Related Dexamethasone Induced 1) encodes a member of the Ras superfamily of small GTPases and is induced by dexamethasone (https://www.genecards.org/cgi-bin/carddisp.pl?gene=RASD1) and is considered to be a tumor suppressor (Vaidyanathan et al., 2004) (Zhao et al., 2019). The PPI network analysis in high vs low MUC1 shows MAPK12 and MAPK10 interacting with each other and both downregulated in high MUC1 samples. AMH is downregulated in high MUC1 samples and is shown clustering with the BMP4 network. RASD1 is downregulated and clusters with CREB3L3 (Supplementary Figure S1).

It is very important to mention here that we only had 29 PDA samples from the TCGA to distinguish based on MUC1 expression levels, out of which only seven were low MUC1. Although all the 30 genes were differentially expressed in low vs high/moderate MUC1 samples with statistical significance, due to the low sample size being a limitation in this particular study, it is difficult to conclude any correlations with certainty. The findings need to be validated with a larger cohort in the future. However, despite the low sample size, we found that MUC1 expression had a significant correlation with poor OS in PDA patients (Figure 1B) confirming its clinical significance as a biomarker yet again. For our downstream analysis, we selected JNK (a component of the MAPK pathway) since MAPK was commonly altered in all the three differentially regulated pathways (MAPK, TGF-β and BMP-4) from the heatmap.

Using a panel of human PDA cell lines, we demonstrated that high-MUC1 expression is positively correlated to TGF-βRII expression (Figure 2B) with a high statistical significance, a receptor that activates the non-canonical pathway. Furthermore, there was a trend of negative correlation between high-MUC1 expression and TGF-βRI expression, albeit not significant, a receptor that activates the canonical SMAD pathway, known to drive cells towards cell death and apoptosis (Valderrama-Carvajal et al., 2002).

If TGF-β mainly activates TGF-β receptor II in high-MUC1 PDA cells, it should lead to increased activation of the non-canonical pathway genes. Accordingly, we found that overexpression of MUC1 in MiaPaca2 cells induced increased phosphorylation of JNK and c-Myc (Figures 3A,B), which signify activation of the non-canonical pathway associated with cellular proliferation and invasion (Li et al., 2015). TGF-β significantly increased phosphorylation of c-Myc at Ser62, which is a marker of stability of c-Myc (Wang et al., 2011). On the other hand, HPAFII treated with control siRNA had high levels of phosphorylated JNK and c-Myc with TGF-β exposure, but when MUC1 was downregulated using a specific siRNA, the phosphorylation of JNK and c-Myc were significantly reduced (Figure 3B), thus, TGF-β destabilized c-Myc when MUC1 expression is low in PDA cells (Figure 3C). Overall, the data show the important contribution of MUC1 in driving the TGF-β mediated non-canonical pathway.

As was expected, TGF-β treatment reduced cell viability in low MUC1 PDA cell line MiaPaca2 but increased cell viability in high MUC1 PDA cell line HPAFII (Figure 4A). However, when MUC1 was overexpressed in MiaPaca2, TGF-β increased the viability of these cells significantly (Figure 4B), and when MUC1 was knocked down in HPAFII cells, TGF-β reduced cell viability (Figure 4C), thus clearly showing MUC1-dependent gain-of-function and loss-of function in TGF-β signaling switch towards a tumor promotor.

If indeed TGF-β signaling is critical for the aggressive growth of high-MUC1 PDA tumors, then neutralizing TGF-β with an antibody *in vivo* would dampen tumor growth. Confirming our hypothesis, neutralizing TGF-β treatment in high-MUC1 HPAFII tumors significantly reduced tumor progression and reduced tumor burden (Figure 5B), whereas the same treatment almost hastened tumor growth in low MUC1 MiaPaca2 tumors (Figure 5B). These data conform with our hypothesis that blocking TGF-β will be beneficial in PDA with high-MUC1 but may aid in tumor growth in low-MUC1 PDA.

**FIGURE 6:**
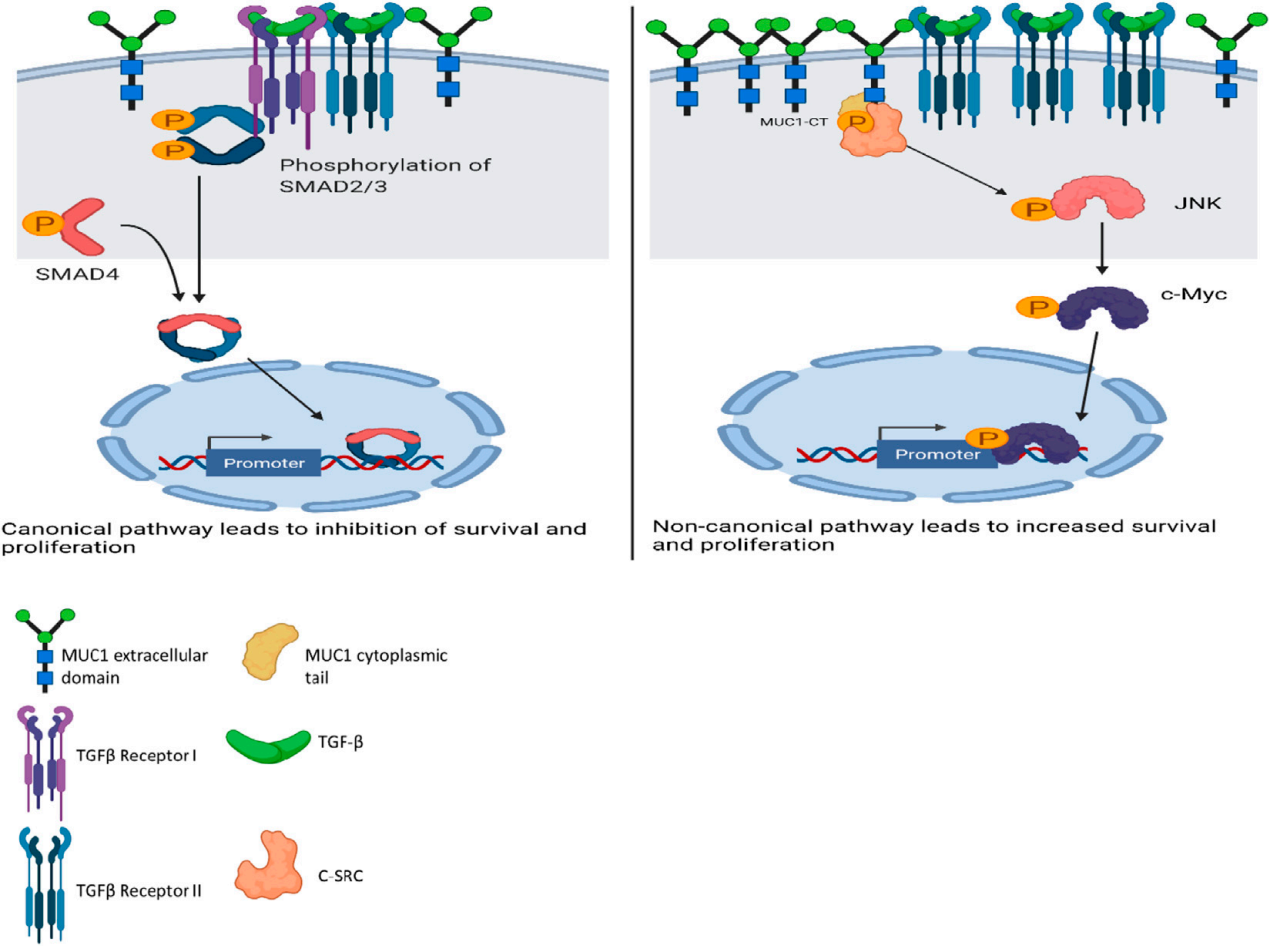
**Schematic diagram of the proposed mechanism of TGF-β signaling and functions in high versus low MUC1 PDA**. Left panel shows activation of SMAD-dependent canonical pathway in low-MUC1 PDA cells. TGF-β ligands bind to the membranous TGF-β receptor (TGF-βRII) homodimers with high affinity. TGF-βRII binding allows dimerization with TGF-β type I receptor (TGF-βRI) homodimers, activation of the TGF-βRI kinase domain and signal transduction via phosphorylation of the C-terminus of receptor-regulated SMADs (R-SMAD), SMAD2 and SMAD3. The SMAD2/3 dimer then forms a heterotrimeric complex with SMAD4 which translocates in the nucleus (Massagué and Wotton, 2000; Ross and Hill, 2008). This leads to growth inhibition, cell cycle arrest and apoptosis of PDA cells, thus TGF-β acts as a tumor suppressor. **Right panel** shows activation of SMAD-independent non-canonical pathway in high-MUC1 PDA cells. In this pathway, binding of TGF-β mainly to TGF-β-RII most likely increases phosphorylation of c-SRC which in turn phosphorylates MAPK, followed by JNK and c-Myc (Bunda et al., 2014). This phosphorylation cascade activates the MAPK/JNK pathway and stabilizes c-Myc which translocates into the nucleus to increase transcription of oncogenic proteins and leads to increased growth, invasion and EMT of PDA cells (Fey et al., 2016). MUC1-CT also aids in the process by its oncogenic signaling. Thus, in high-MUC1 PDA cells TGF-β acts as a pro-tumorigenic cytokine. The schematic was created with BioRender.com.

A schematic diagram illustrates our current understanding of MUC1’s role in switching TGF-β signaling from a canonical tumor suppressive to a non-canonical tumor promoting pathway (Figure 6).

The data has uncovered a major role of MUC1 in regulating the paradoxical function of TGF-β in PDA. To the best of our knowledge, this is the first report that shows significant changes in gene expression profiles in the TGF-β, MAPK and BMP signaling pathways in patient-derived RNA-seq samples from PDA, based solely on MUC1 expression levels. These data indicate the clinical relevance of MUC1 in modulating the TGF-β signaling in PDA. In addition to the bioinformatics data, we report significant correlation of MUC1 to TGF-βRII protein expression levels in a panel of human PDA cell lines which informs the downstream signaling in response to TGF-β. Thus, TGF-β activates the non-canonical JNK pathway in the high-MUC1 PDA cells (which also express higher TGF-βRII). While, in the low MUC1 cells (that express higher levels of TGF-βRI), TGF-β reduces viability and inhibits growth possibly leading to apoptosis. 

Finally, our study also shows that PDAs with high-MUC1 are more likely to respond to anti-TGF-β therapy but PDAs with low-MUC1 will probably have poorer prognosis with the same treatment. Therefore, MUC1 expression may be used as a surrogate biomarker to determine the efficacy of future TGF-β-targeted treatments for PDA and possibly other gastrointestinal cancers. Thus, we suggest that MUC1 expression may be used as a biomarker to personalize the treatment with TGF-β targeted treatment modalities. We recognize that further studies need to be performed to elucidate the causal relationships between MUC1 and the other differentially expressed genes in the TGF-β pathway, however, using genetically identical PDA cells that had MUC1 knocked down or MUC1 overexpressed, we have addressed the causal relationship between MUC1 and TGF-β signaling.

## Materials and Methods

### TCGA Gene Expression Analysis

Twenty-nine pancreatic adenocarcinoma tumor RNA-Seq data were downloaded from the Genomic Data Commons data portal (Grossman et al., 2016). All tumor samples were from the PAAD project data generated by The Cancer Genome Atlas (TCGA) Research Network: http://cancergenome.nih.gov/. The tumor samples were separated in two groups based on their MUC1 expression: MUC1 low expression group and MUC1 moderate/high expression group. Seven tumor samples had extremely low MUC1 expression values. HTSeq-counts data was input into DESeq2 (version 1.32.0) to identify differentially expressed genes in MUC1 moderate/high vs MUC1 low expression samples (Love et al., 2016). Genes with an adjusted *p*-value < 0.05 and log2 fold change difference greater than two were considered differentially expressed. Gene set enrichment analysis was performed with all the DEGs. The enrichR package in R was used to identify enriched gene sets from Gene Ontology (GO) Biological Process, Molecular Function, and Cellular Component (2021) and the KEGG database (2021) (Kuleshov et al., 2016). The top 10 sets were collected from each database. There are a few pathways of specific interest in this study: the MAPK, BMP, and TGF-beta signaling pathway. DEGs were filtered to only include those that are involved in at least one of these pathways. Thirty genes in these three pathways were differentially expressed and used for further analysis. A heatmap was created with these pathway DEGs, using pheatmap (version 1.0.12) package in R. To further visualize the effects of MUC1 expression, only low and high MUC1 expressed samples were included (samples with moderate MUC1 expression were excluded). DESeq2 analysis was conducted with only these samples and a protein-protein interaction (PPI) network was created from the DEGs, using the STRING database (1.7.0). The list of DEGs were input into the STRING protein query to create a PPI network for the significant genes. The STRING database is a collection of known and predicted protein-protein interactions identified from multiple types of sources (Szklarczyk et al., 2021). The identified PPI network was visualized in Cytoscape (version 3.9.0) (Shannon et al., 2003) with the color of the nodes representing the gene log fold change value. The Kaplan-Meier plot was generated by calculating the survival curve using the survival package (3.1–8) in R and visualized using the survminer (0.4.9) package in R.

### Cell Lines and Culture

Human PDA cell lines (CFPAC, HPAC, HPAF-II, Panc1, MiaPaCa2, Su86.86 and BxPc3) were obtained from American Type Culture Collection and cultured as instructed. Cell lines were maintained in Dulbecco’s Modified Eagle Medium (DMEM; Gibco), Minimal Essential Media (MEM; Gibco), or Roswell Park Memorial Institute 1,640 medium (RPMI; with, l-glutamine; ThermoFisher). All media was supplemented with 10% fetal bovine serum (FBS; Gibco or Hyclone), 3.4 mM l-glutamine, 90 units (U) per ml penicillin, 90 ug/ml streptomycin, and 1% Non-essential amino acids (Cellgro). Cells were kept in a 5% CO_2_ atmosphere at 37°C. MUC1 WT sequence was cloned into the pLNCX.1 vector consisting of the neomycin resistance gene (n*eo*) and confirmed by DNA sequencing. MiaPaca2. MUC1 and MiaPaca2. Neo were generated by transfection with Lipofectamine 3,000 (Thermo Fisher) according to the manufacturer’s protocol and maintained in medium containing Geneticin (G418; Invitrogen, Carlsbad, CA, USA) (Roy et al., 2011). Neo cells had the empty vector with the G418 resistance gene (*neo*) and MUC1 cells had the full length MUC1 gene and G418 resistance gene (*neo*). Every passage of MiaPaca2 transfected cells were maintained in a final concentration of 150 μg/ml of the antibiotic G418 (50 mg/ml) (Thermo Fisher) to ensure positive selection. HPAFII cells were serum-starved for 24 h and then treated with control siRNA from Life Technologies or MUC1 siRNA from Perkin Horizon according to the respective manufacturer’s protocol using Lipofectamine RNAiMAX Transfection Reagent (Thermo Fisher Scientific) for 72 h, followed by treatment with TGF-β. For all experiments, cell lines were passaged no more than 10 times.

### Treatment With TGF-β and Western Blotting

The cell lines used were MiaPaca2. Neo, MiaPaca2. MUC1, HFAFII. controlsiRNA, and HPAFII. MUC1siRNA. Cells were serum starved for 48 h and treated with either 10 ng/ml of human TGF-β (Peprotech, Rocky Hill, NJ, USA) or the vehicle (citrate buffer) for 10 min. HPAFIICell lysates were prepared and western blotting performed as previously described (Roy et al., 2011). Membranes were blocked with commercial blocking buffer (Thermo Fisher) for 30 min at room temperature and incubated with primary antibodies overnight at 4°C. The antibodies used were: Armenian hamster monoclonal anti-human MUC1 cytoplasmic tail (CT2) antibody (1:500). MUC1 CT antibody CT2 was originally generated at Mayo Clinic and purchased from Neomarkers, Inc. (Portsmouth, NH) (Schroeder et al., 2001). CT2 antibody recognizes the last 17 amino acids (SSLSYNTPAVAATSANL) of the cytoplasmic tail (CT) of human MUC1. Membranes were also probed with the following antibodies from Cell Signaling Technology (1:1,000), p-JNK, total JNK, β-Actin (Mouse, 3,700), p-c-Myc (Ser62) (Invitrogen), total c-Myc (Invitrogen). Other antibodies used include TGF-βRI (Abcam, 1:200, Rabbit, ab31013) and TGF-βRII (Abcam, 1:1,000, Rabbit, ab61213). Densitometric analysis was conducted using the ImageJ software and percent change was calculated accordingly. First, each density unit for the particular protein was normalized to their respective β-actin density and then represented as phospho/total.

### MTT Assay

5,000 cells were plated in 96 well plates and allowed to grow overnight. After serum starvation for 24 h, the cells were treated either with control buffer or 10 ng/ml of TGF-β in triplicates for 24–96 h. Then 20ul of MTT solution (5 mg/ml) was added to each well and incubated for 3–4 h at 37°C. Following that, the media with MTT was removed and 200ul of DMSO was added to each well to dissolve the formazan crystals for 10 min and the O.D. was measured with a plate reader (Multiskan, Thermo Fisher) at 560 nm.

### Xenograft Studies

Athymic Nude-Foxn1nu mice were purchased from Harlan Laboratories and housed at UNC Charlotte’s vivarium. These mice were injected subcutaneously with tumor cells. 3 × 10^6^ HPAFII cells (50ul) (n = 9) or 5 × 10^6^ MiaPaCa2 cells (50ul) (n = 12) were injected with Matrigel (50ul) (total = 100ul) subcutaneously into the flank of male or female Athymic Nude-Foxn1nu mice (Murphy et al., 2012). Once the tumors reached a palpable size (∼3 × 3mm, ∼5 days post tumor inoculation), mice were separated into four different groups. Groups 1 and 2 had HPAF-II tumors and groups 3 and 4 had MiaPaca2 tumors. Groups 1 and 3 were treated with the isotype control IgG antibody (20ug/100ul per mouse) three times a week for 2 weeks. Groups 2 and 4 were treated with the monoclonal TGF-β neutralizing antibody (LifeTech) (20ug/100ul per mouse) three times a week for 2 weeks. Mice were monitored daily for general health and tumors were palpated. Caliper measurements were taken three times a week over 28 days until endpoint and once euthanized, tumor wet weight was taken (tumor size: ∼15 × 15 mm) (Figure 5A). This study and all procedures were performed after approval from the Institutional Animal Care and Use Committee of UNC Charlotte.

### Immunohistochemistry

For nonenzymatic antigen retrieval, sections were heated to 85°C in Dako antigen retrieval solution for 90 min and cooled for 20 min; all subsequent steps occurred at room temperature. To quench endogenous peroxidase, slides were rinsed and incubated in methanol/2% H_2_O_2_ for 10 min. Sections were then washed, blocked in 50% fetal bovine serum (FBS) in PBS for 45 min, and incubated overnight with primary antibodies. Sections were incubated for 1 h with secondary antibody, developed with a diaminobenzidine (DAB) substrate (Vector Inc., Burlingame, CA, USA), counterstained with hematoxylin, and mounted with Permount. Primary antibodies used were Armenian hamster anti-MUC1 cytoplasmic tail (CT), CT2 (1:50) and anti-TGF-β antibody (Novus Biologicals) (1:10). Secondary antibodies used were rat anti-hamster HRP conjugated antibody (1:100, Jackson Labs) and anti-mouse HRP conjugated antibody (Cell Signaling Technology) (1:50). IgG conjugated to horseradish peroxidase was used as negative control. Immunopositivity was assessed using light microscopy and images taken at 100× magnification.

### Statistical Analysis

The data are expressed as the mean±SEM of n = 3. Differences between groups were examined using unpaired two-tailed t-tests, one-way and two-way ANOVAs. Statistical comparisons were made using the GraphPad Prism 9.0. *p*-values of <0.05 were considered statistically significant (*p <  0.05; **p  <  0.01; ***p < 0.001; ****p < 0.0001).

## Data Availability

Publicly available datasets were analyzed in this study. This data can be found here: The TCGA PAAD publicly available gene expression dataset was analyzed in this study. The data can be found at the Genomic Data Commons Data Portal: https://portal.gdc.cancer.gov/repository?facetTab=files&amp;filters=%7B%22op%22%3A%22and%22%2C%22content%22%3A%5B%7B%22content%22%3A%7B%22field%22%3A%22cases.case_id%22%2C%22value%22%3A%5B%22set_id%3ALHoP3XwBj5K9rWEJJI1I%22%5D%7D%2C%22op%22%3A%22IN%22%7D%5D%7.
