# Supplementary material for: Overexpression of MUC1 Induces Non-Canonical TGF-β Signaling in Pancreatic Ductal Adenocarcinoma
Source: Front Cell Dev Biol. 2022 Feb 14;10:821875. doi: 10.3389/fcell.2022.821875 (PMC8883581; doi:10.3389/fcell.2022.821875)

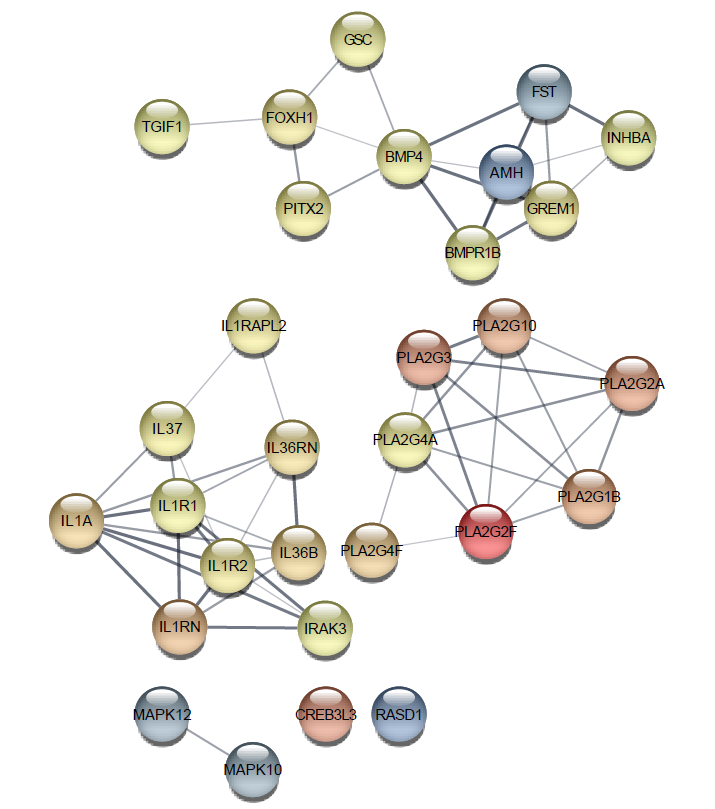


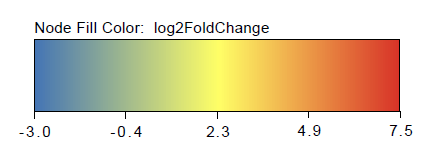


**Figure S1. The Protein-protein-interaction network as determined by STRING and visualized in Cytoscape for the 30 genes in the TGF-β, MAPK and BMP4 pathways.**


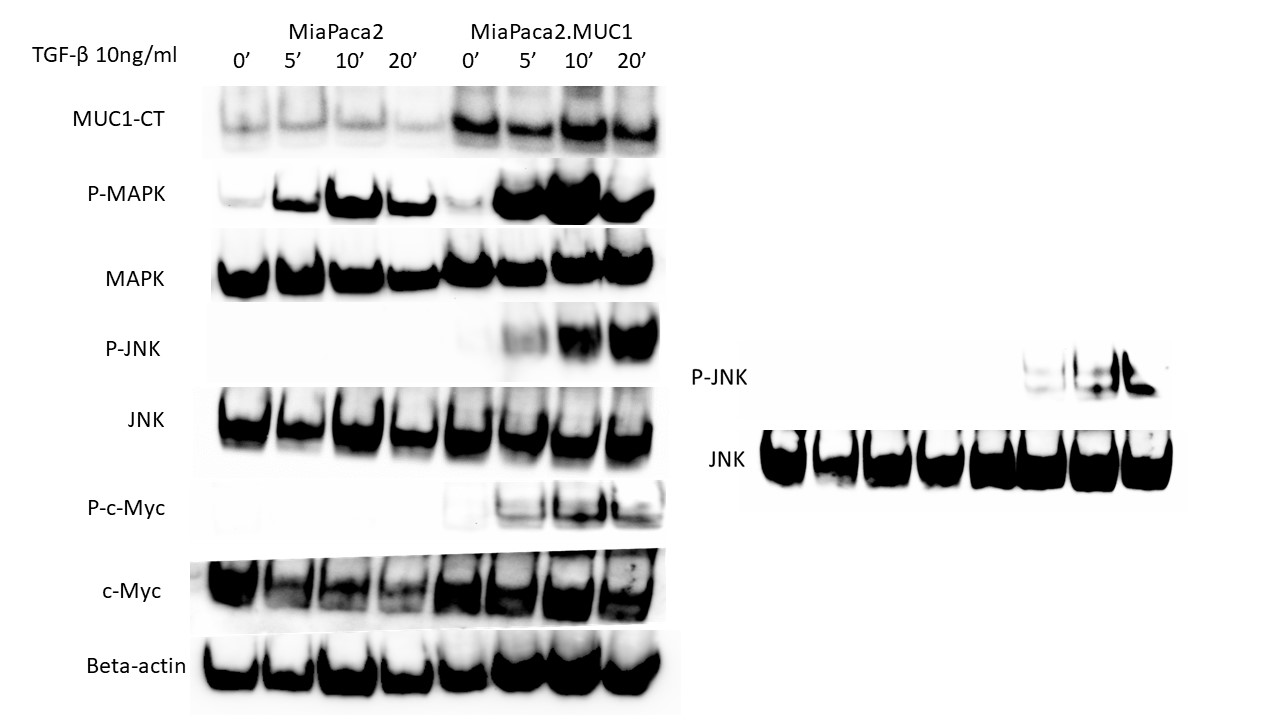


**Figure S2. Overexpression of MUC1 leads to increased phosphorylation of MAPK, JNK and c-Myc**. Western blot expression of phosphorylation of MAPK, JNK and c-Myc compared to total MAPK, total JNK and total c-Myc in MiaPaca2 vs MiaPaca2.MUC1 cells in response to 10ng/ml of TGF-β at 0, 5, 10 and 20 minutes. β-actin was used as endogenous loading control.


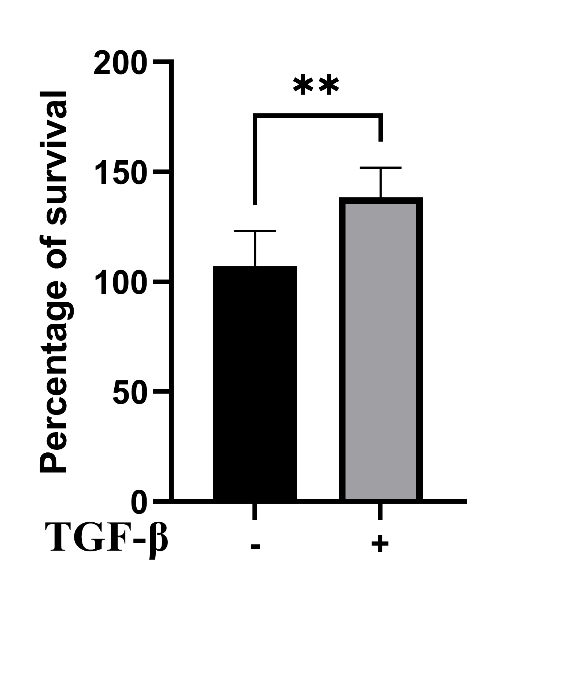

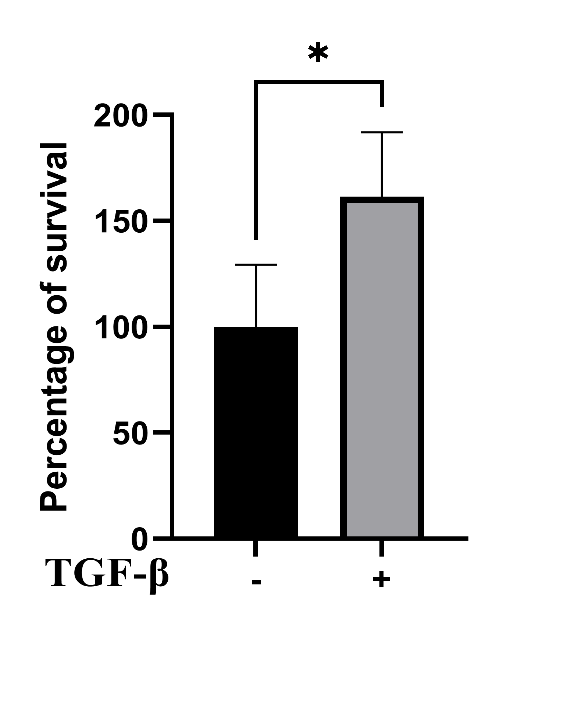


B

A

**Figure S3.** **TGF-β exposure increases viability in cells with high MUC1.** MTT cell viability assay on **A.** HPAFII and **B** MiaPaca2.MUC1 cells with 10ng/ml of TGF-β for 96 hours. All data are shown as means +/− SEM of n=3. Unpaired t-test was performed to compare between treated and untreated cells for all experiments. * p < 0.05, ** p < 0.01, *** p<0.001, **** p<0.0001.


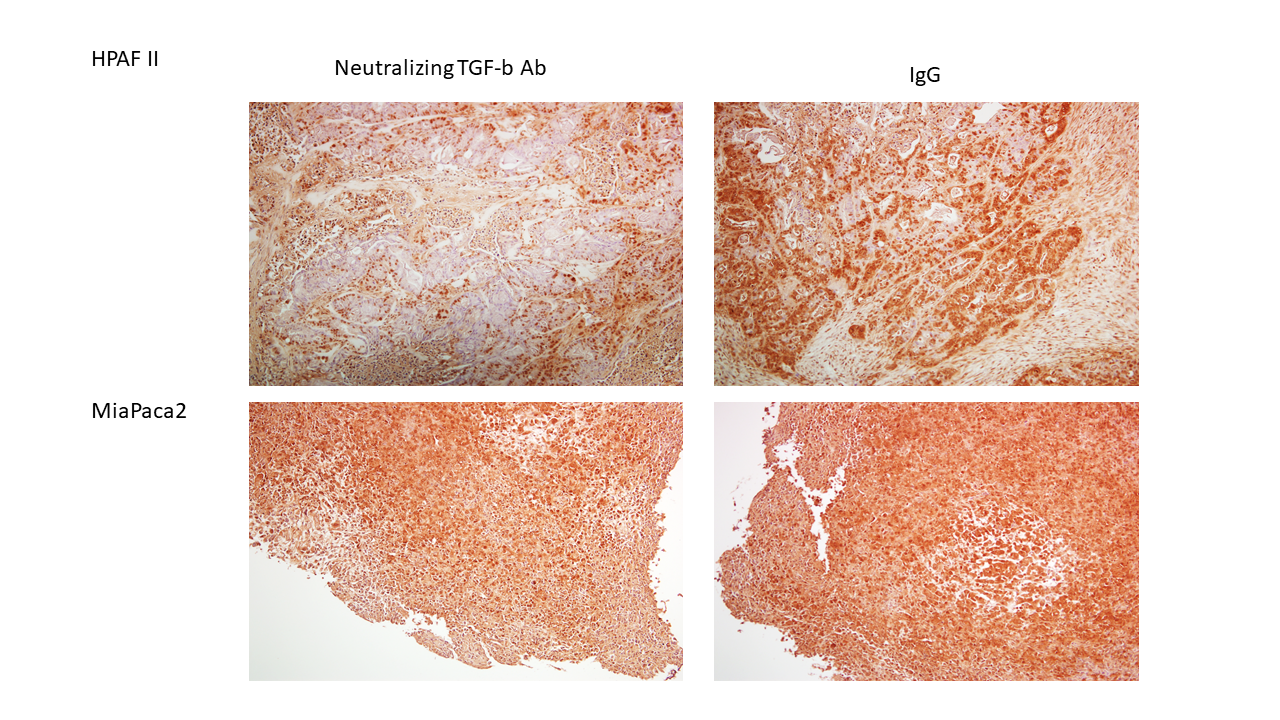

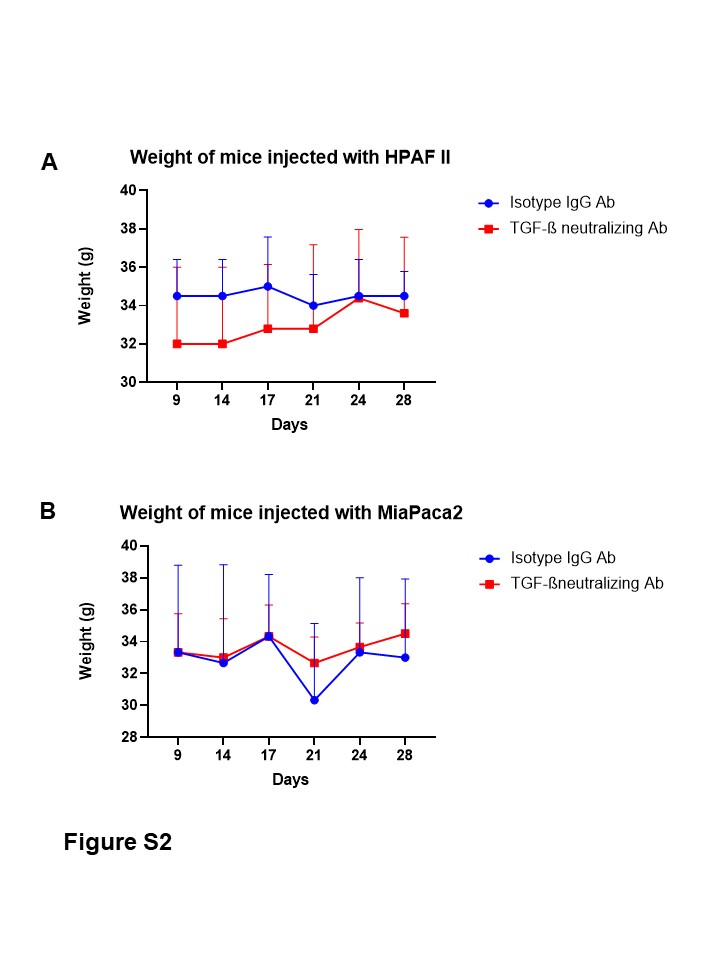


MiaPaca2

HPAFII

A

B

**Figure S4. A. Immunohistochemistry for TGF-β.** Top. HPAFII tumor tissues and Bottom. MiaPaca2 tumor tissues showing TGF-β expression in mice treated with neutralizing TGF-β antibody (left) and isotype control IgG (right). **B. Body weights of all mice remained consistent over the period of the in vivo study.** Body weights of all nude mice injected with Top. HPAFII cells and Bottom. MiaPaca2 cells and treated with IgG isotype antibody (blue) and TGF-β neutralizing antibody (red) over the period of 28 days starting from the day of treatment are shown.

**Supplementary Table 1. Table showing the characteristics of the 29 PDA samples from TCGA.**


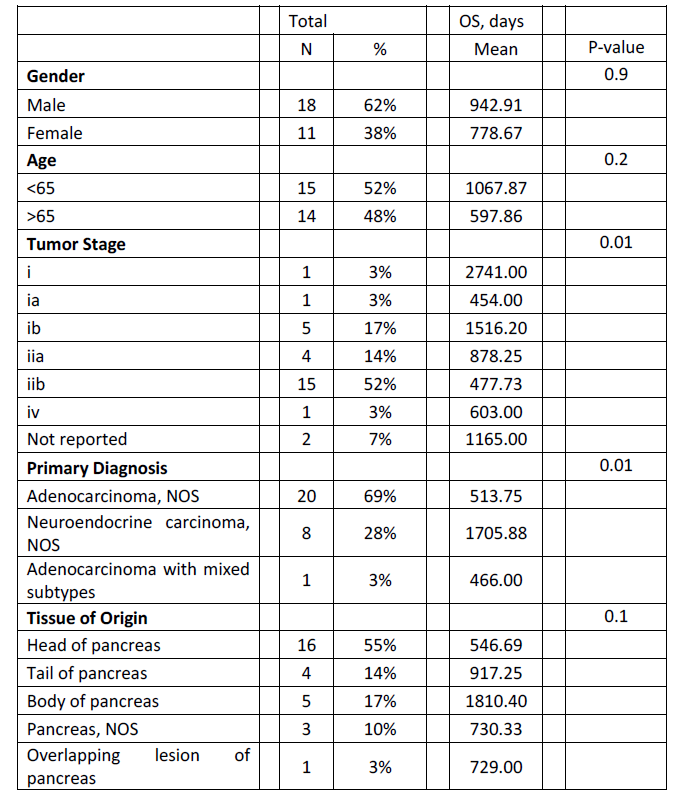

Supplement: Supplementary file 2 [file DataSheet2.docx]
