# Supplementary material for: Overexpression of MUC1 Induces Non-Canonical TGF-β Signaling in Pancreatic Ductal Adenocarcinoma
Source: Front Cell Dev Biol. 2022 Feb 14;10:821875. doi: 10.3389/fcell.2022.821875 (PMC8883581; doi:10.3389/fcell.2022.821875)

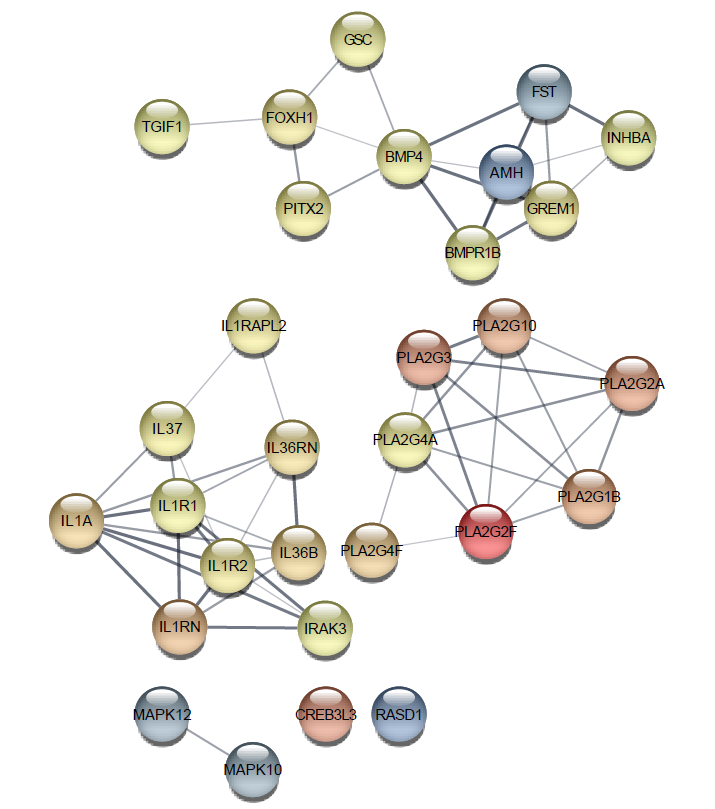


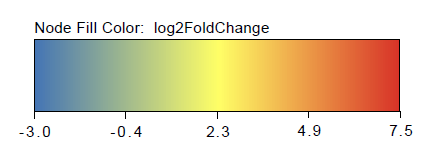


**Figure S1. The Protein-protein-interaction network as determined by STRING and visualized in Cytoscape for the 30 genes in the TGF-β, MAPK and BMP4 pathways.** Protein-Protein Interaction (PPI) network of the top 3 clusters from the 30 genes in high vs low MUC1 samples are shown. The color of the nodes in the PPI network represents the log (FC) value of the gene expression in the different groups. The size of nodes reflects the number of interacting proteins with the designated ones. The edge width indicates the combined scores obtained from STRING. The figure was constructed by Cytoscape (v3.6.1).


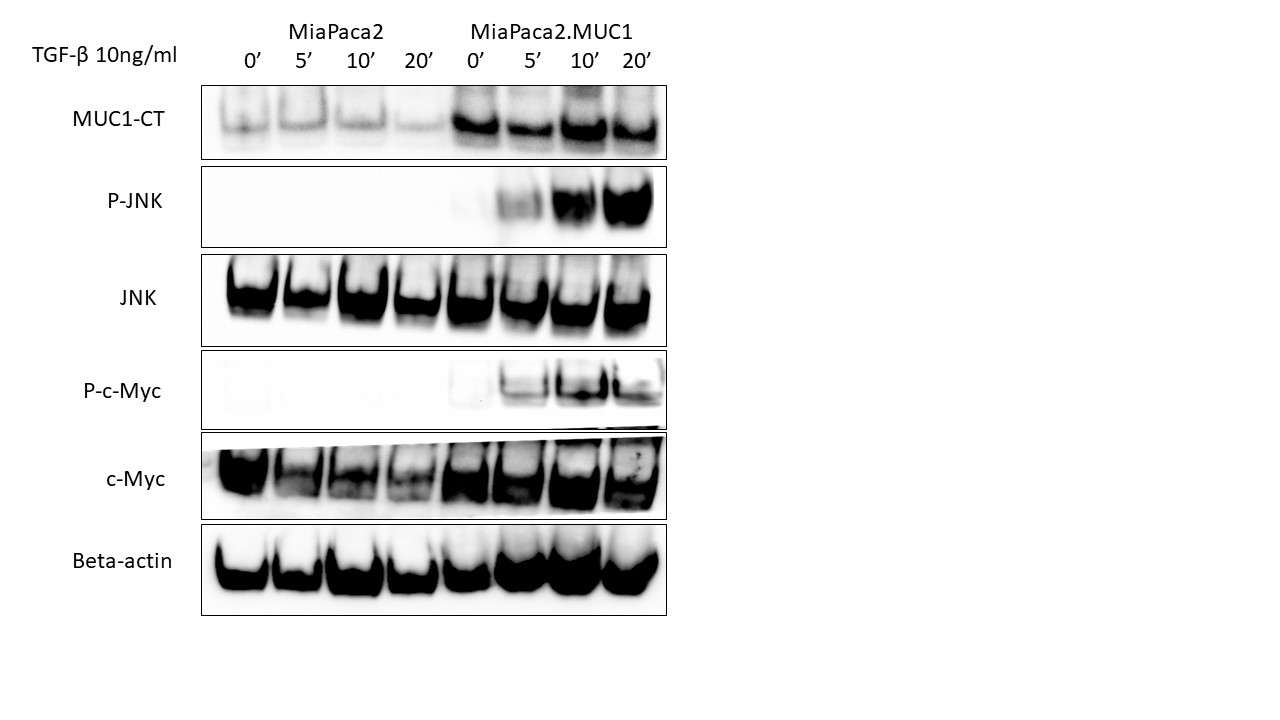


**Figure S2. Overexpression of MUC1 leads to increased phosphorylation of JNK and c-Myc**. Western blot expression of phosphorylation of JNK and c-Myc compared to total JNK and total c-Myc in MiaPaca2 vs MiaPaca2.MUC1 cells in response to 10ng/ml of TGF-β at 0, 5, 10 and 20 minutes. β-actin was used as endogenous loading control.


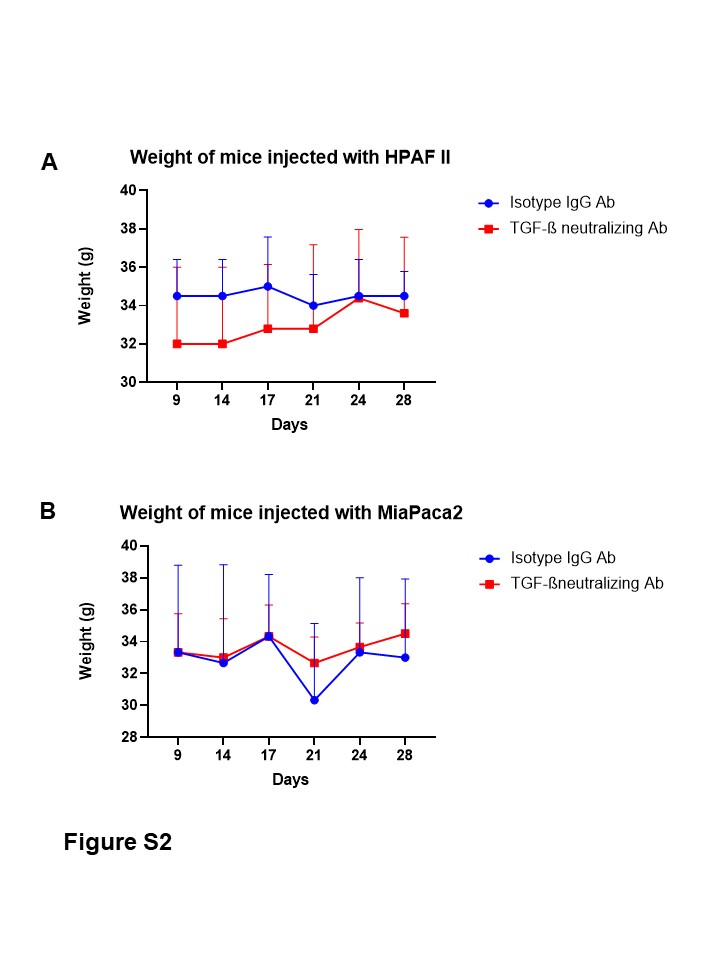


**C**


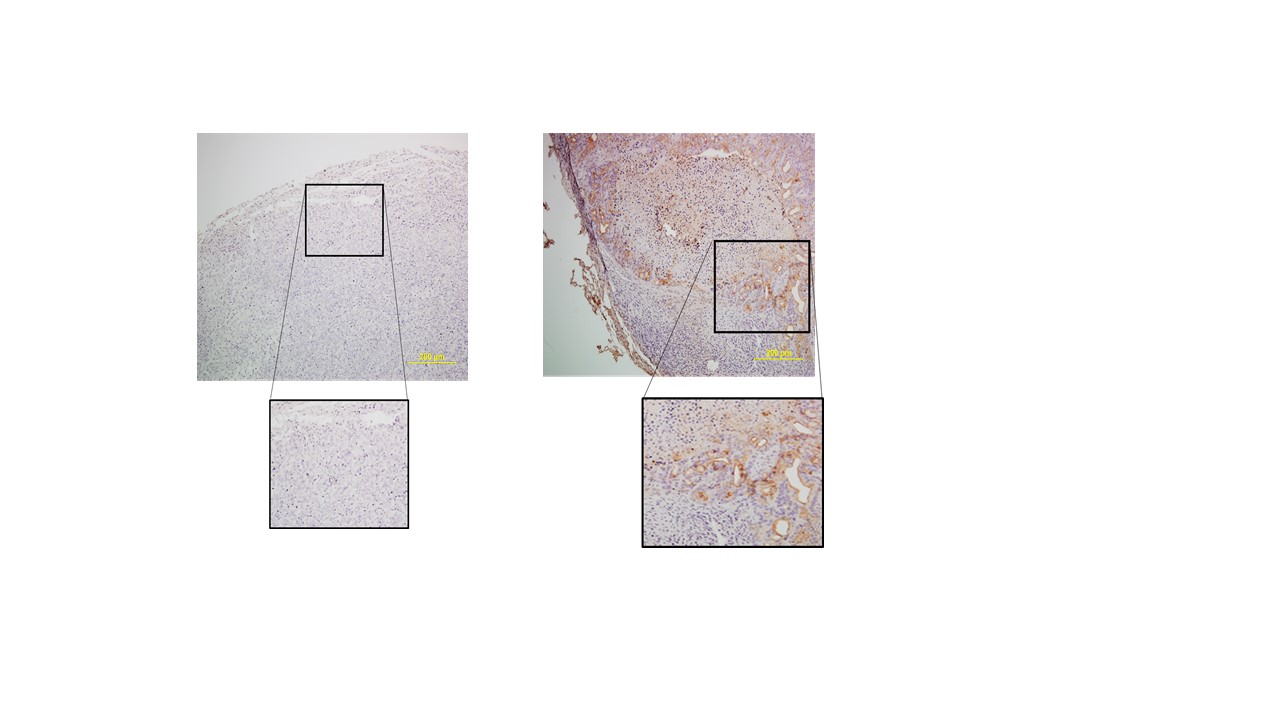


**Supplementary Figure 3. Body weights of all mice remained consistent over the period of the in vivo study.** Body weights of all nude mice injected with A. HPAF II cells and B. MiaPaca2 cells and treated with IgG isotype antibody (blue) and TGF-β neutralizing antibody (red) over the period of 28 days starting from the day of treatment are shown. **C.** Immunohistochemistry showing expression of MUC1 in MiaPaca2 (left) and HPAF II (right) tumors.

**Supplementary Table 1. Table showing the characteristics of the 29 PDA samples from TCGA.**


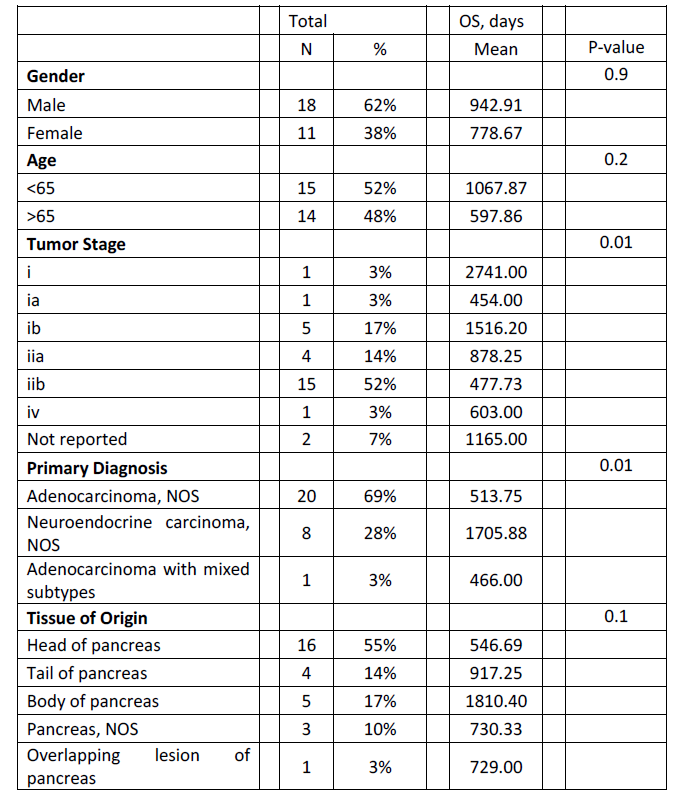

Supplement: Supplementary file 3 [file DataSheet1.docx]
